# Biomaterials for organically generated habitats beyond Earth

**DOI:** 10.1126/sciadv.adp4985

**Published:** 2025-07-02

**Authors:** Robin Wordsworth, Rafid Quayum, Elida Kocharian, Ann Pearson, Xavier Portillo, Madeleine Yang, Charles S. Cockell, Shannon Nangle, George Church

**Affiliations:** ^1^ School of Engineering and Applied Sciences, Harvard University, Cambridge, MA 02138, USA.; ^2^ Department of Earth and Planetary Sciences, Harvard University, Cambridge, MA 02138, USA.; ^3^ Center for Astrophysics, Harvard University, Cambridge, MA 02138, USA.; ^4^ Department of Genetics, Harvard Medical School, Harvard University, Boston, MA 02115, USA.; ^5^ School of Physics and Astronomy, University of Edinburgh, Scotland, UK.; ^6^ Circe, Boston, MA, USA.

## Abstract

Sustaining life beyond Earth requires the creation of habitats, which is typically assumed to require costly transport of high-mass components from Earth. Here, we investigate an alternative approach based on in situ fabrication using biologically generated materials. We show that several common biomaterials are capable of blocking UV radiation, transmitting visible light, and maintaining pressure differences sufficient to permanently stabilize liquid H_2_O in a vacuum or low-pressure environment. As a proof of concept, we then demonstrate growth of eukaryotic green alga in a 3D printed PLA bioplastic habitat under Mars-relevant conditions of a 600 Pa CO_2_ background atmosphere. Our results demonstrate that products of biology itself can be used to create habitats in extraterrestrial environments. This approach is scalable, sustainable, and plausibly could be extended to construction of human habitats in the future.

## Teaser

Bioplastics can be used to manufacture sustainable extraterrestrial habitats.

## INTRODUCTION

Earth is the only place in the cosmos known to support life. The basic requirements of carbon-based life are a source of free energy, liquid water, and access to bio-essential elements (minimally C, H, N, O, P, and S) (*1*). Beyond Earth, habitable environments to life in the solar system are thought to exist in the interiors of icy moons and possibly on extremely limited regions of Mars, but they are otherwise rare (*2*, *3*).

In the near future, human technology will enable the expansion of life beyond Earth. Any sustainable long-term human presence in space will require high levels of in situ resource utilization (ISRU) (*4*, *5*). Increasingly, it is becoming clear that biotechnology has the potential to play a pivotal role in ISRU (*6*–*11*). Primary producers (plants, green algae, and cyanobacteria) can provide critical resources such as food, structural materials, and chemical fuels (*12*–*16*) and enable the extraction of raw materials from their surroundings (*13*, *17*). Just as human life on Earth is sustained by the biosphere, any long-duration human activity beyond Earth will require the support of an ecosystem.

**Fig. 1.**
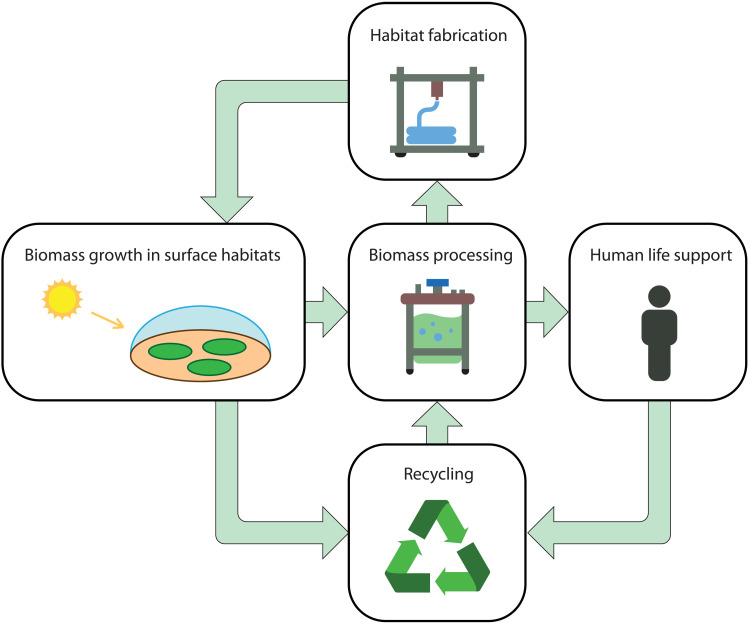
Schematic of the biomaterials approach to life support in extraterrestrial environments. In this approach, raw materials for habitat construction are synthesized from algal and plant feedstocks. Biomass growth, processing, and recycling all take place in surface habitats made from recyclable bioplastics.

Key advantages of biotechnology include scalability, robustness, and the ability to recycle waste materials efficiently. However, existing concepts for space biotechnology generally rely on industrially manufactured habitats. For photosynthetic organisms such as plants or algae, this typically means growth in indoor or underground bioreactors using artificial electric lighting powered by an external source (solar or nuclear). While effective on small scales, this approach is power-intensive and limited by available habitat space. If transparent surface habitats (*18*) could instead be constructed from biologically generated materials, it would be a major step forward, as it would allow self-sustaining generation of biomass for life support in extraterrestrial environments with minimal power requirements (Fig. 1).

To sustain photosynthetic life, a habitat must transmit sufficient photosynthetically active radiation, block harmful ultraviolet (UV) radiation, and maintain pressure and temperature in a range that allows the presence of liquid water. Pressure is a particularly important limitation. Mars has an average atmospheric pressure of ~600 Pa with spatial and temporal variations of several 100 Pa, while on the Moon and asteroids, atmospheric pressure is below 1 μPa (*19*, *20*).

In the 10° to 30°C, temperature range optimal for algal growth, the saturation vapor pressure of H_2_O is 1.2 to 4.2 kPa. Past studies of bacterial growth under low-pressure conditions indicate viability down to around this limit, as long as sufficient nutrients are available (*21*, *22*), with limited data available for eukaryotic organisms (*23*). Desirable properties of biomaterials for habitat construction therefore include high transmittance of visible radiation and low transmittance of UV, low H_2_O permeability, and strength sufficient to maintain a pressure gradient of several kilopascals. Additional considerations include the ease of habitat manufacture, resistance to degradation by environmental chemicals and UV radiation, and potential for recycling waste materials.

## RESULTS

### Bioplastic material properties

Common commercial bioplastics include agarose, polylactic acid (PLA) and polyhydroxyalkanoates (PHAs). Agarose, a heteropolysaccharide derived from red algae, is straightforward to synthesize from commercially available feedstocks. However, it is permeable to H_2_O, soluble in liquid water and brittle when dry. PLA, a thermoplastic derived from condensation of lactic acid, is now the most widely produced bioplastic (*24*). It exhibits low degradation rates when exposed to UV light. Last, PHAs are a diverse group of polyesters that are produced via bacterial fermentation. They are also UV-stable but are more biodegradable than PLA (*25*, *26*).

We measured key physical properties of samples of these materials (Table 1). Measured yield strengths varied from about 3 to 55 MPa. Using scaling based on the hoop stress equation, *Y* ~pr/t , this indicates wall thicknesses of less than *t* = 1 mm are required to sustain a pressure difference of *p* = 10 kPa in a habitat with radius *r* = 10 cm. Our 1-mm-thick test samples completely blocked the most harmful UV-C radiation (100 to 280 nm) and partially blocked UV-A and -B radiation (280 to 400 nm). The transmission of visible radiation was high in the agarose and translucent PLA samples but negligible in the PHA sample. Based on all these factors, we selected the PLA for additional testing.

**Table 1.** Experimentally determined optical and physical properties of several common bioplastics. Here, $\kappa_{\text{vis}}$ is the visible extinction coefficient, $\kappa_{\text{UV}-\text{AB}}$ is the UV-A/B extinction coefficient (280 to 400 nm), and σ is the average ultimate stress. All samples fully blocked UV-C radiation. Errors shown are 1-σ SD. See Materials and Methods for details on our experimental approach.

Biomaterial	$\kappa_{\text{vis}}$ (mm^−1^)	$\kappa_{\text{UV}-\text{AB}}$ (mm^−1^)	σ(MPa)
Agarose	0.54 ± 0.28	1.93 ± 1.02	9.84 ± 5.71
Translucent PLA	0.65 ± 0.06	0.94 ± 0.10	27.9 ± 5.74
PHA	>2.2	>3.4	9.16 ± 1.89

### Algal growth experiments

**Fig. 2.**
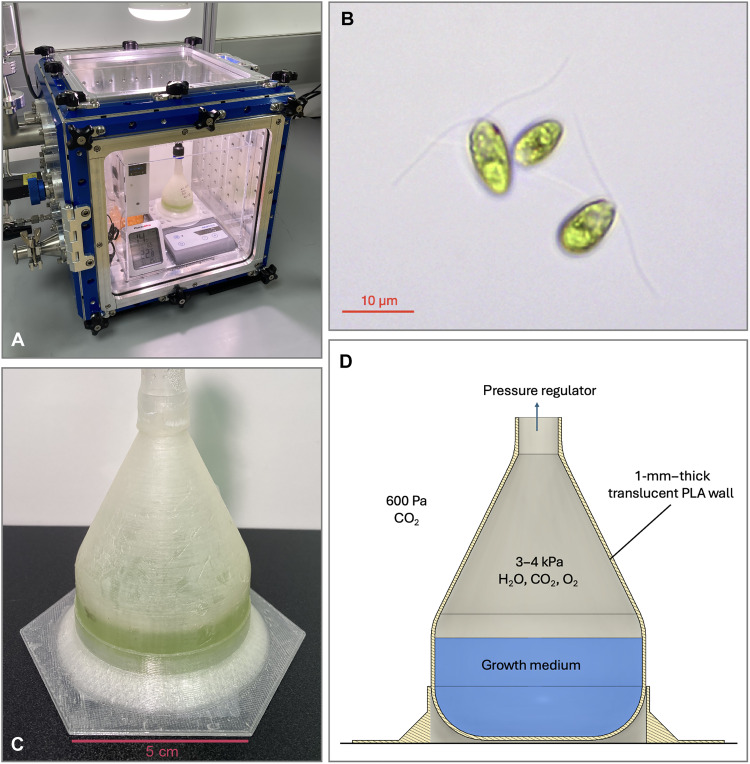
Overview of our algal growth experiments. (**A**) Image of the bioplastic habitat inside the planetary environment chamber during an experiment, (**B**) optical microscope image of *D. tertiolecta* strain used in the growth experiments, (**C**) close-up image of the bioplastic habitat, and (**D**) schematic of the habitat inside the planetary environment chamber.

To demonstrate the feasibility of growing photosynthetic organisms in biologically generated habitats, we performed experiments in a Mars analog 600-Pa CO_2_ atmosphere (Fig. 2). We chose Mars as a test case as it presents a major habitability challenge compared to Earth but is less hostile than most other extraterrestrial environments. Our experimental setup consisted of a pressure-regulated planetary environment chamber, with a CO_2_ inlet valve controlled by a mass flow regulator. A solenoid valve and pressure gauge attached to a desktop computer were used to maintain an internal pressure of 600 ± 20 Pa. A residual gas analyzer (RGA) was used to monitor gas composition in the chamber. Under normal operating conditions the gas mixture was >98% CO_2_.

For our proof of concept experiment, we fabricated a small bioplastic habitat using a Dremel 3D45 printer, with translucent PLA as the working material (Fig. 2). The wall thickness was 1 mm. Conventional additive manufacturing does not produce airtight structures, so we sealed the walls of the chamber with a 50:50 mixture of organic wax and resin (primarily palmitic, stearic, and abietic acid). Our light source was a light-emitting diode (LED) bulb with flux of 12.45 W m^-2^ onto the outer surface of the habitat (measured using a double-dome pyranometer). The flux inside the habitat was 3.6 W m^−2^, so the visible light attenuation factor was 71%. For comparison, the mean solar flux at the surface of Mars in mid-latitude locations is around 150 W m^−2^, while that at Ceres is around 44.4 W m^−2^, indicating that light levels are not a limiting factor for photosynthesis in the inner solar system.

Our chosen algal species was *Dunaliella tertiolecta* (Figure 2). *Dunaliella* is a motile eukaryotic green alga that is used as a feedstock for aquaculture and has been extensively studied for its biotechnology applications (*27*). The algae were grown in Erdscheiber’s medium (*28*), while the recorded temperature in the habitat was 23 ± 1°C. A differential pressure regulator was used to keep the pressure difference between the bioplastic habitat interior and its surroundings below 5 kPa. Pressures inside the habitat during growth experiments were 3–4 kPa, with the gas composition a mixture of H_2_O, CO_2_ and O_2_. We recorded algal growth by sampling from the habitat in two-day intervals and counting the cell density via hemocytometry.

**Fig. 3.**
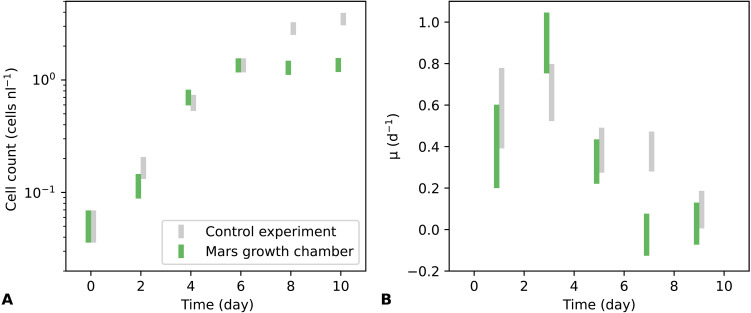
Results of the algal growth experiments. (**A**) Cell population and (**B**) growth rate parameter μ versus time for growth experiments conducted in the bioplastic habitat surrounded by a 600-Pa CO_2_ atmosphere (green) and a control experiment performed under ambient conditions (gray).

**Fig. 4.**
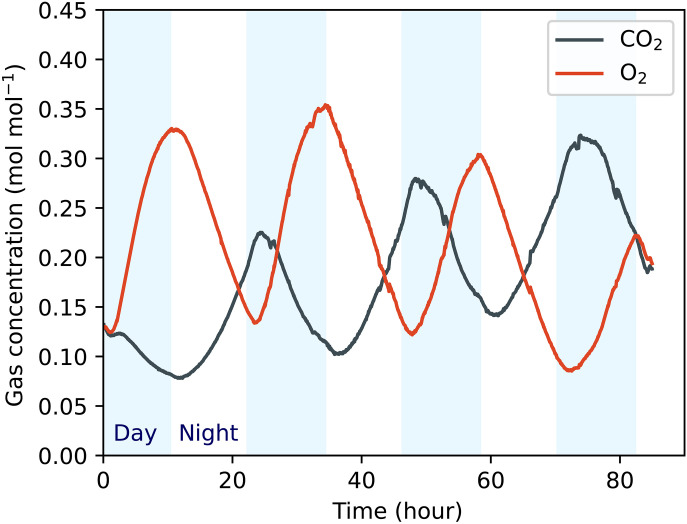
Evidence for photosynthesis and respiration in the bioplastic habitat. CO_2_ and O_2_ concentrations as a function of time are shown, as measured by the RGA. Measurements were started after the 10 days of growth recorded in Fig. 3. Blue shading indicates periods when the lamp was switched on.

Figure 3 shows results for *Dunaliella tertiolecta* growth in the bioplastic habitat over 10 days with a Mars analog background 600 Pa CO_2_ atmosphere in the simulation chamber and 12:12 day-night cycle, as well as a control experiment performed at ambient pressure (1 atm) with the same culture volume and surface area. Robust growth, close to that achieved in the control experiment, was observed under simulated Mars conditions. Growth rate (μ, day^−1^) peaked at around 0.9 day^−1^ after ~3 days. As an additional check, we used the RGA to observe changes in CO_2_ and O_2_ inside the bioplastic habitat for 3.5 days, starting after 10 days of growth (Fig. 4). Variation in gas concentration was observed due to alternating cycles of daytime photosynthesis and night-time respiration. We also studied the size and morphology of the algae after completion of the growth phase and did not observe any significant differences from the control experiment. Finite-difference estimation of the time derivative of O_2_ from the data in Fig. 4 revealed peak O_2_ release rates of around 5 μmol hour^−1^. Given the measured cell density of around 1 cells nl^−1^ from hemocytometry (Fig. 3) and a final medium volume of 43.0 ml, this implies a photosynthesis rate of ~120 fmol O_2_ cell^−1^ hour^−1^, comparable to measured literature values under more typical conditions (*29*, *30*).

### pH and CO_2_ permeability

**Fig. 5.**
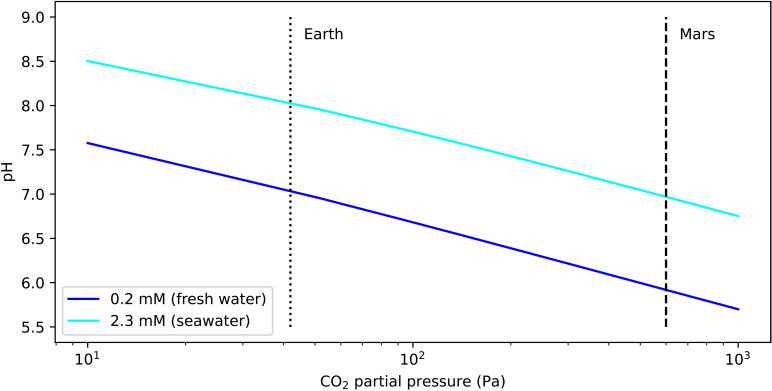
CO_2_ decreases pH, which can affect habitability for some microbial species. Plot shows pH as a function of CO_2_ partial pressure for fresh water and terrestrial seawater. For comparison, the partial pressures of CO_2_ in the atmospheres of Earth and Mars are shown.

The growth medium pH decreases with the partial pressure of CO_2_ at a rate dependent on the alkalinity of the medium. Figure 5 shows pH modeled as a function of CO_2_ partial pressure for seawater alkalinity (2.34 mM) and, for comparison, low-alkalinity freshwater (0.2 mM). The seawater curve intersects Earth’s CO_2_ partial pressure of 42 Pa at pH = 8.1, while at Martian $p_{{\text{CO}}_{2}}$ the value is 7.0. The reduced buffering capacity of freshwater yields much lower pH values of 7.0 and 5.9, respectively. Growth of *D. tertiolecta*, here in the seawater medium, was not strongly affected by the decreased pH in the planetary environment chamber experiments. For more sensitive species, deliberately increasing the alkalinity of the growth medium would be one way to manage pH under elevated CO_2_ conditions.

**Fig. 6.**
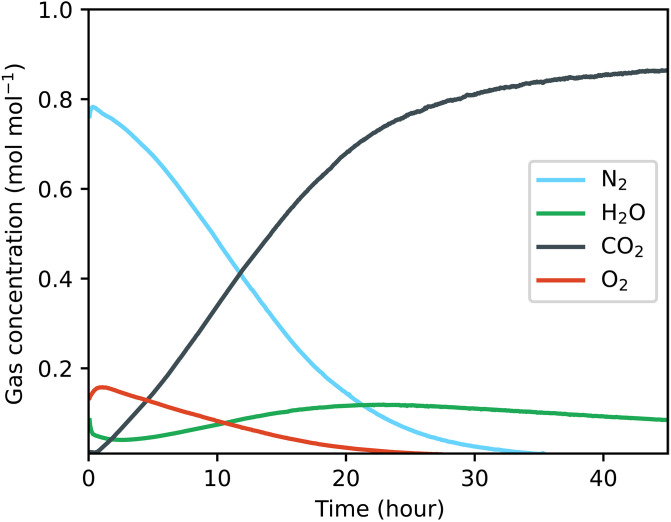
The habitat gas permeability was determined from RGA measurements. Plot shows change in gas composition inside the dry bioplastic habitat after initiation of low-pressure conditions in the planetary environment chamber (600-Pa CO_2_).

Carbon was not a limiting nutrient for growth in our experiments because the bioplastic habitat walls were permeable to CO_2_. We performed a dry test in the planetary environment chamber to determine the habitat permeability (Fig. 6) and observed that CO_2_ partial pressure inside the habitat took ~0.5 days to reach a steady-state value when the chamber pressure was 600 Pa. This implies an effective permeability (*31*) of around *P* ~ tV/(ARTτ)=230 barrer [7.8 × 10^−14^ mol m/(m^2^ s Pa)], where τ = 0.5 days; *R* is the universal gas constant; temperature *T* = 23°C; and the habitat wall thickness, surface area, and volume are *t* = 0.001 m, *A* = 0.0132 m^2^, and *V* = 1.1 × 10^−4^ m^3^, respectively. Measured *P* values for CO_2_ transport through PLA are lower than this (*32*), so slow leaks through the three-dimensional (3D) printed habitat wall likely dominated gas exchange.

In a real application on Mars, the optimal choice of wall permeability would involve a trade-off between permitting CO_2_/O_2_ exchange and minimizing H_2_O loss. Because CO_2_ and O_2_ are far less polar than H_2_O, nonpolar hydrophobic barriers that inhibit H_2_O diffusion would facilitate this. For other extraterrestrial locations such as the Moon or deep space, near-total inhibition of gas exchange would be desirable, which could be achieved via a combination of thicker habitat walls and the use of nanocomposites to decrease gas diffusion (*33*).

### Temperature regulation, radiation, and toxicity

We have focused on how the low pressure challenge to habitability in extraterrestrial environments can be overcome, but other challenges exist. Temperature was held constant in our experiments, but Mars, the Moon, and other extraterrestrial environments exhibit large temperature variations. For example, typical diurnal mean values on Mars are from 170 to 225 K in mid-latitude regions where near-surface ice is present (*34*, *35*). Low-temperature environmental conditions can be overcome via internal heating, which requires an external power source, or via the solid-state greenhouse effect, which can passively cause temperature increases of 50 K or more with silica or nanocellulose aerogel layers a few cm thick (*35*–*37*). Nanocellulose is generated from biological feedstocks, which would allow temperature control to be integrated into the closed-loop framework of Fig. 1. Similar to PLA and PHA bioplastics, aerogels can be deposited using additive manufacturing techniques (*38*).

Ionizing radiation from solar events and galactic cosmic rays, while hazardous to humans, is much less of a concern for plants and algae (*39*). On Mars, the toxicity of perchlorate in near-surface regolith is an additional challenge (*40*). However, it can be mitigated via microbial perchlorate reduction (*41*), sourcing essential nutrients from the subsurface, or potentially genetic engineering of perchlorate-resistant plant species (*42*, *43*).

### Scalability of closed-loop biomass production

A detailed techno-economic study of our biology-centered concept for life support is beyond the scope of this paper, but its basic feasibility can be demonstrated by the following calculation. If an extraterrestrial habitat uses a bioplastic barrier of thickness *t* = 1 mm, then the barrier mass per unit area is 1.25 kg m^−2^ given typical bioplastic density ρ ~ 1250 kg m^−3^. A 2-cm nanocellulose layer of density 69 kg m^−3^ (*37*) to provide solid-state greenhouse warming of 50 K or more (*35*) has mass per unit area of 1.4 kg m^−2^.

**Fig. 7.**
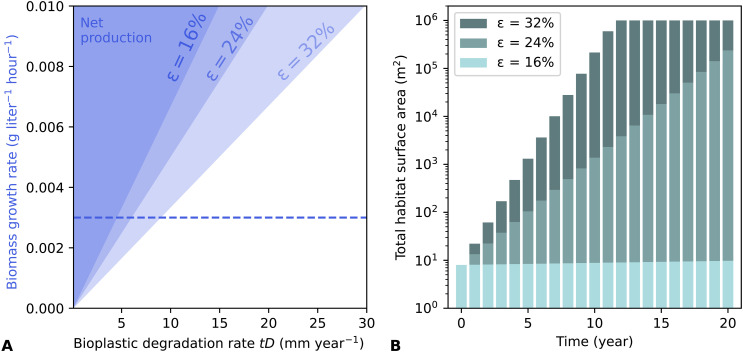
Bioplastic production rates above a break-even threshold enable exponential growth of habitat area. (**A**) Growth rates for which habitat area increases over time as a function of the bioplastic degradation rate, for three different values of the bioplastic conversion efficiency ϵ . Shaded regions show where net growth occurs, and dashed line indicates the biomass growth rate in our experiments, for comparison. (**B**) Simulated growth of habitat area based on (*1*), given three different efficiency values for biomass to bioplastic conversion. In this example, growth is halted once an area of 1 km^2^ is reached. Calculation uses numbers given in the main text for other variables and assumes no other restrictions on growth.

Taking the lifetime of the barrier to be just 1 year implies that the average rate of bioplastic production must be equal to or greater than 3.4 g m^−2^ day^−1^. If cellulose production for temperature control is also required, this implies an additional ~3.8 g m^−2^ day^−1^. A peak cell density of ~1 cell nl^−1^ close to that in our experiment (which was not optimized for growth) yields a productivity of $\dot{q}=0.003$ g liter^−1^ hour^−1^, assuming a growth rate of μ = 0.5 day^−1^ and mean cell dry mass of 150 pg (*44*). A habitat depth *d* = 0.3 m with average productivity of 0.003 g liter^−1^ hour^−1^ (*45*) would produce 22 g m^−2^ day^−1^, so with efficiency in conversion of biomass to bioplastic of ϵ=16% , net production would be achieved (Fig. 7A). Cellulose production for temperature control has a similar required efficiency of 18%. These conversion efficiencies are low compared to typical values achieved in terrestrial production facilities (*46*).

Excess bioplastic production could be used for human life support purposes, to expand the total habitat area, or both. The latter use would lead to exponential growth in habitat area in the absence of other limiting factors. This can be quantified by writing an expression for the rate of change of habitat area with time

$$\frac{\mathrm{dA}}{\mathrm{dt}}=A\left(\epsilon G-D\right)$$ (1)

where *G* and *D* are the rates of biomass growth and bioplastic degradation, respectively, in units of year^−1^. The rate of production of bioplastic per unit area is $\epsilon \dot{q}d$ . Expressed as a fraction of the habitat wall mass per unit area ρt , this gives $\epsilon G=\epsilon \dot{q}d/\rho t$ . Growth occurs when ϵG>D (Fig. 7A). Given a starting area $A_{0}$ , the equation solution is $A=A_{0}e^{\left(\epsilon G-D\right)t}$ (Fig. 7B). Depending on ϵ , doubling time of habitat area can be as low as 1 to 2 years, resulting in large increases in habitat area over a 20-year period in the absence of other bottlenecks on growth. Power requirements would be minimal compared to a traditional bioreactor because both light and heat are supplied directly by the environment. Another advantage of this approach is that degraded wall material could be recycled back to usable nutrients in bacterial bioreactors, further increasing efficiency and decreasing waste (Fig. 1).

## DISCUSSION

We have demonstrated that habitable conditions can be maintained in extraterrestrial environments using only biologically produced materials. Our work is in part inspired by the remarkable adaptability of life on Earth to diverse environments. Because the biology-driven route to sustaining life beyond Earth has several distinct advantages over purely industrial approaches, there are a number of interesting implications for future research.

In the near-term, it would be interesting to study a wider range of species under varying atmospheric conditions. We have used *Dunaliella* as a model organism, but our results suggest that it should be possible to grow any similar photosynthetic organism as long as elemental availability, light, and pH conditions are met. PLA can be produced via the synthesis of sugars from CO_2_ and H_2_O, followed by fermentation to lactic acid and then polymerization in the presence of a catalyst (*47*). Sugars can be produced by photosynthetic algae, while fermentation is performed by bacteria such as *Lactobacillus*. The third step typically requires heating to the 150° to 210°C range in the presence of metal catalysts such as tin octoate (C_16_H_30_O_4_Sn) but potentially could also be performed by biological enzymes such as lipase (*48*). PHA, in contrast, is produced as intracellular granules in bacterial species such as *Cupriavidus necator*, allowing direct mechanical or enzymatic extraction after growth. This makes PHA a promising bioplastic to study for extraterrestrial applications in the future.

Synthetic biology offers rich additional possibilities for in situ resource utilization as it provides ways to increase performance and resilience in hostile, resource-limited environments. Modifying photosynthetic pathways could boost production of oxygen, while enhancements to carbon fixation pathways or up-regulation of key enzymes involved in lipid synthesis could boost the production of organic materials, including PLA feedstocks or PHAs. Future progress in this area will need to focus on refining regulatory mechanisms to improve production of vital compounds. By leveraging adaptive evolutionary strategies and precision genetic modifications, systems could be designed to self-regulate in response to environmental and metabolic cues.

Investigation of additional ideas for stabilizing liquid water in low-pressure environments would be interesting. One alternative to bioplastic barriers is compression, which can be achieved by placing a layer of hydrophobic liquid of sufficient depth over liquid water. A typical triglyceride oil layer with density of around 920 kg m^−3^ and a depth of 1 m will cause a pressure difference at the oil-water interface of 3.4 kPa, given the hydrostatic relation *p* = ρ*gh* and martian gravity *g* = 3.72 m s^−2^. The mass requirements for such a solution are much higher than with a bioplastic barrier, but this might be mitigated by the simplicity of producing oils from microbial or plant feedstocks.

We have shown here how biomaterials can create habitable environments for photosynthetic life, but the biology-driven habitability concept is readily extendable to construction of human habitats. Humans are more sensitive to pressure and fluctuations in gas composition and are vulnerable to solar and galactic cosmic radiation, but all of these challenges can plausibly be addressed using organically sourced materials. As an additional benefit, further research on the use of biodegradable materials for extraterrestrial construction should also advance sustainability objectives on Earth, given the ongoing global crisis in plastic pollution (*49*, *50*).

Although industrial methods often outperform biological systems in terms of raw efficiency in isolated applications, the diverse advantages of biotechnology mean that it will play a central role in supporting life in space in the future. Extremophile microbes are adept at growing under a wide range of conditions, but the stability of liquid water places hard limits on habitability for microscopic organisms. Using the natural products of biology to enable closed-loop habitability on a larger scale allows these limits to be overcome. The results reported here represent an important step forward, but many additional steps are needed to enable robust ecosystems to be sustained long-term beyond Earth.

## MATERIALS AND METHODS

Translucent PLA samples were printed using Dremel filament and printer model 3D45. We used an Original PRUSA MK4 3D printer to fabricate samples of PHA using filament from ColorFabb, a company that manufactures 100% biodegradable PHA filament. The agarose bioplastic was created in batches by mixing 600 ml of distilled water with 15 g of commercial food-grade agar powder (Living Jin brand) and 10 ml of glycerol. The resulting mixture was heated to 100°C for 20 min, sieved, and then poured into silicone molds, where it was left to cool to room temperature. The resulting hydrogels were then desiccated in an electric oven at 50°C for 48 hours, after which they were cut, weighed, and measured.

Bioplastic visible light and UV bioplastic transmittances were measured using a double glass dome pyranometer (Hukseflux Instruments model SR-11) and calibration-certified Sper Scientific UV-A/B and UV-C detectors, respectively. The UV-A/B detector had peak sensitivity in the 350- to 360-nm range, with 365-nm calibration point. The UV-C detector had peak sensitivity in the 255-to 265-nm range, with 254-nm calibration point. Calibration errors of the pyranometer and UV detectors were ±1 and ±4%, respectively. The UV transmission experiments were performed using a compact 4-W UV lamp with dual tubes to emit radiation peaking in either the 365-nm (UV-A/B) or 254-nm (UV-C) range. For the visible light source, a General Electric BR30 LED bulb was used. Bioplastic strength measurements were performed using an Instron single-column strength testing machine. Strain tests were performed until each sample fractured and surpassed its ultimate stress value. Sample thickness and length values were measured using precision calipers and a metal rule. Errors in radiance, thickness, and length were determined from repeat measurements and propagated using a linear Gaussian model.

The experimental apparatus for the biological growth experiments is depicted in fig. S1. A vacuum chamber consisting of a 12-by-12-by-12–inch volume modular cube (Ideal Vacuum) was connected to a mass flow regulator (Sierra SmartTrak 50) that supplied CO_2_ at a constant flow rate (default value of 20 sccm/min). Windows on the chamber allowed transmission of light from an external source and monitoring of the interior during experiments. A rotary vane pump (Pfeiffer Pascal 2021SD) attached to a solenoid valve was used to control the chamber pressure in the 10 Pa to 100-kPa range. Chamber pressure was measured by a piezo transducer gauge (MKS Instruments 902B). A desktop computer connected to several ATmega328P microcontrollers was used to control the apparatus and collect data.

The chamber gas composition was measured using a mass spectrometer (Stanford Research Systems RGA 100). The spectrometer was attached to a turbopump (Pfeiffer HiCube 80) to bring down pressure to around 1 × 10^−4^ Pa for ion probe operation. A natural-light LED bulb (General Electric BR30) was placed above the chamber as the algae light source. Broadband visible flux from the lamp at the top of the bioplastic habitat was measured using the Hukseflux SR-11 pyranometer.

The bioplastic habitat was designed in Autodesk Fusion 360 and fabricated using the 3D45 model Dremel 3D printer. Translucent PLA was used as the working material. To avoid damage to the bioplastic habitat during initiation of the experiments, it was connected to a custom-built differential pressure regulator. This regulator consisted of a small pressure sensor connected to a solenoid valve, with an Arduino controller programmed to open the valve for differential pressures greater than 5 kPa. Typical pressures inside the habitat during growth experiments were 3 to 4 kPa. The partial pressure of H_2_O at 23°C is 2.8 kPa (*51*). As a result, the headspace gas mixture in the habitat was dominated by H_2_O when liquid water was present, with the remainder a mixture of CO_2_, O_2_, and other minor species.

We modeled pH in the habitat medium using the software package PyCO2SYS. Erdschreiber’s medium is derived from near-surface seawater, for which an alkalinity value of 2300 mM is appropriate (*52*). We verified that pH remained in the 7 to 9 range for seawater under Mars conditions via a colorimetric test in the simulation chamber.

*D. tertiolecta* microalgae purchased from UTEX supplies (strain LB 999) was cultured using sterile technique in Erdschreiber’s medium, which was also purchased from UTEX. The control experiment was incubated at 23°C in an autoclaved 125-ml Erlenmeyer flask. At the beginning of the experiment, 100 ml of Erdschreiber’s medium was innoculated with 1 ml of a *D. tertiolecta* sample in the exponential growth phase, yielding an initial cell density of ~0.05 cells nl^−1^. Fifty microliters of the innoculated medium was then added separately to the control experiment flask and the bioplastic habitat.

Cell counting was conducted every 2 days using an optical microscope with ×10 magnification eyepiece and ×10 objective. Twenty microliters of fluid from the habitat was pipetted onto a disposable hemocytometry slide with Neubauer Improved ruling pattern (purchased from Nexcelom Bioscience). Cells were counted over a total area of 4 mm^2^, and the result was divided by the total volume (400 nl) to yield cell number density in units of cells per nanoliter (Fig. 3A). We corrected for evaporative losses by weighing the habitat and Erlenmeyer flask after each measurement. Typical mass loss was 0.2 to 0.7 g between measurements.

The growth rate parameter μ in Fig. 3B was calculated by finite difference from the cell density data as $\mu =\left(\text{log}N_{i+1}-\text{log}N_{i}\right)/\Delta t$ , where Δ*t* = 2 days is the time interval between measurements and $N_{i}$ is the measured cell number density at time $t_{i}$ . The error in μ was calculated as

$$\sigma_{\mu }=\sqrt{{\left.\frac{\sigma_{N}}{N\Delta t}\right|}_{i}^{2}+{\left.\frac{\sigma_{N}}{N\Delta t}\right|}_{i+1}^{2}}$$ (2)

The error in *N* was calculated as

$$\sigma_{N}=\sqrt{N}+0.1N$$ (3)

where the first term is the Poisson (counting) error and the second term is included to account for systematic effects such as cell clumping.

The photosynthetic O_2_ production rate in the habitat was calculated using the ideal gas law and differentiation of the data in Fig. 6 (see also fig. S2). The total volume of headspace air in the habitat and attached tubing was *V* = 109.6 ml. The measured habitat pressure when the RGA data was recorded was *p* = 3.2 kPa and temperature was *T* = 23°C. We used the ideal gas law $n_{O_{2}}=p_{O_{2}}V/\mathrm{RT}$ to calculate moles of O_2_, where $p_{O_{2}}=c_{O_{2}}p$ , $c_{O_{2}}$ is measured O_2_ molar concentration as plotted in Fig. 6, and *R* is the ideal gas constant. The time derivative ${\mathrm{dn}}_{O_{2}}/\mathrm{dt}$ was then calculated from $n_{O_{2}}$ via numerical differentiation following gaussian filtering with a sigma value of 200 points.

Permeability was calculated by noting that for a gas species *i* with loss flux $J_{i}={\mathrm{Pp}}_{i}/t$ across the habitat wall, where $p_{i}$ is the boundary pressure difference, the timescale for loss of interior pressure is τ ~ $N_{i}/|{\mathrm{dN}}_{i}/\mathrm{dt}|$ , where $N_{i}$ is the total number of moles in the habitat and ${\mathrm{dN}}_{i}\mathrm{dt}=-{\mathrm{AJ}}_{i}$ is the loss rate in mol s^−1^ (*31*, *53*). By the ideal gas law $p_{i}V=N_{i}\mathrm{RT}$ , with the variables defined in the main text. Hence, τ ~ tV/ARTP.
