# Supplementary material for: Biomaterials for organically generated habitats beyond Earth
Source: Sci Adv. 2025 Jul 2;11(27):eadp4985. doi: 10.1126/sciadv.adp4985 (PMC12219502; doi:10.1126/sciadv.adp4985)
Supplement: Supplementary file 1 — Figs. S1 and S2 [file sciadv.adp4985_sm.pdf]

Science Advances
AAAS

Supplementary Materials for

Biomaterials for organically generated habitats beyond Earth

Robin Wordsworth *et al.*

Corresponding author: Robin Wordsworth, rwordsworth@seas.harvard.edu

Sci. Adv. **11**, eadp4985 (2025)
DOI: 10.1126/sciadv.adp4985

This PDF file includes:

Figs. S1 and S2

Supplementary Materials includes two figures, eight python scripts and six data files. The figures show a schematic of the experimental apparatus and a plot of the time rate of change of O_2 in the bioplastic chamber. The python scripts and data files contain all code and data necessary to reproduce the plots.

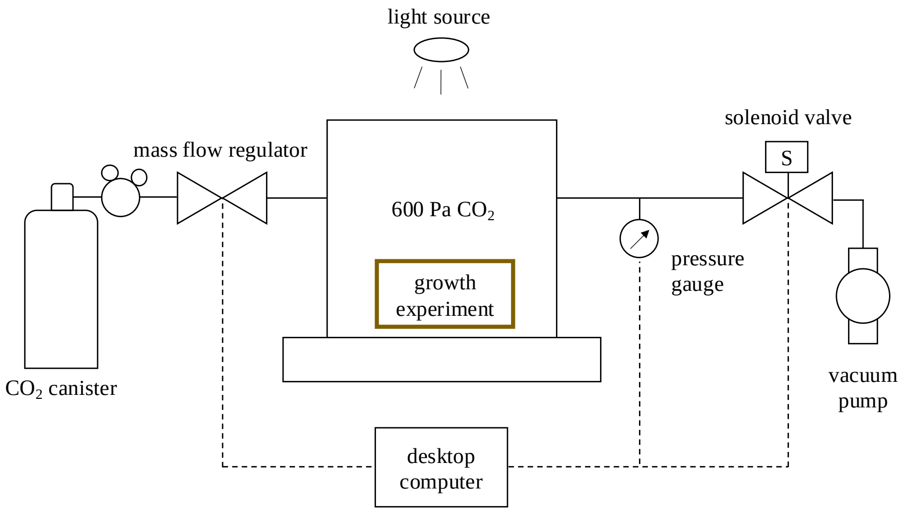

Figure S1: **Schematic of the experimental apparatus.** Solid lines indicate vacuum connections while dashed lines indicates data acquisition and/or active control.

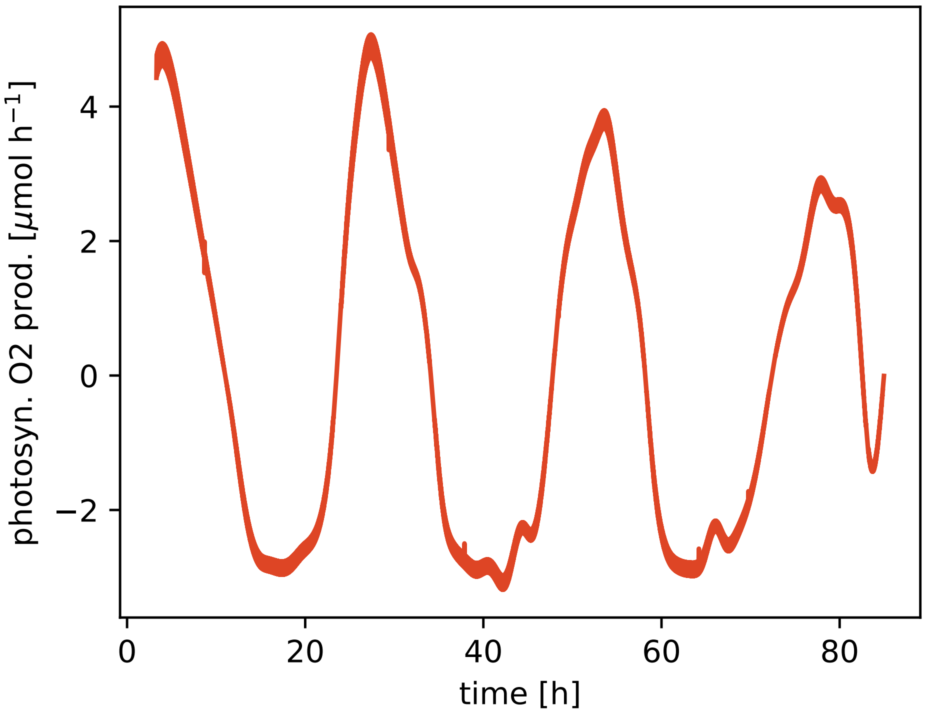

Figure S2: **Variation in chamber oxygen time derivative over time**. Plot of time rate of change of O_2 in the bioplastic chamber, as derived from the data shown in Figure 6.
